# Coexistence of Trisomy 8 and 13 in a Newly Diagnosed Patient With Diffuse Large B Cell Non-Hodgkin Lymphoma and Acute Myeloid Leukemia Secondary to Primary Myelofibrosis

**DOI:** 10.7759/cureus.22217

**Published:** 2022-02-14

**Authors:** Horia Bumbea, Viola Maria Popov, Ciprian Tomuleasa, Meilin Omer, Camelia Dobrea, Irina Manea, Sabina Zurac, Cristiana Popp, Ion Dumitru, Madalina Simoiu, Bogdan Mastalier

**Affiliations:** 1 Hematology, Emergency University Hospital Bucharest, Bucharest, ROU; 2 Hematology, Colentina Clinical Hospital, Bucharest, ROU; 3 Hematology, Chiricuta Oncology Institute, Cluj Napoca, ROU; 4 Morphopathology, Fundeni Clinical Institute, Bucharest, ROU; 5 Radiology, Colentina Clinical Hospital, Bucharest, ROU; 6 Morphopathology, Colentina Clinical Hospital, Bucharest, ROU; 7 Anatomy, Colentina Clinical Hospital, Bucharest, ROU; 8 Hematology, Emergency University Hospital, Bucharest, ROU; 9 Infectious Disease, Matei Bals Institute, Bucharest, ROU; 10 Surgery, Colentina Clinical Hospital, Bucharest, ROU

**Keywords:** trisomia13, trisomia 8, venetoclax, difuse large b cell lymphoma, acute myeloid leukemia

## Abstract

Concomitant diagnosis of non-Hodgkin lymphoma (NHL) and acute myeloid leukemia secondary to chronic myeloproliferative neoplasms (MPNs) is rarely reported. Patients with MPNs may have a second neoplasm, and the risk of lymphoid line neoplasms is 2.5-3.5 times for lymphoid line neoplasms. The explanation for this association is the genetic instability of hematopoietic progenitors in MPNs. An 80-year-old Caucasian man, with many comorbidities, presents for physical asthenia, sweating. The right inguinal adenopathy was known one month before the examination. The patient was diagnosed concomitantly with diffuse large B cell lymphoma (DLBCL) and acute myeloid leukemia (AML) secondary to primary myelofibrosis (PMF) and presented trisomy 8, trisomy 13, and triple-negative PMF status. The patient initially received two well-tolerated R mini CHOP series. This type of treatment was selected to treat DLBCL for one unfit patient for intensive chemotherapy due to his age and comorbidities. R mini CHOP administration was followed by severe aplasia that lasted approximately two weeks followed by severe thrombocytosis that reached 4000 x109/L, and Thromboreductin recommendation was mandatory. The result of the treatment was a partial response but with severe adverse events like neutropenia G4, due to the delay of the treatment the patient lost the response. It was mandatory to select another treatment line and the chosen was venetoclax; it was selected for the simultaneous treatment of DLBCL and the underlying AML. It was obtained a significant reduction in the size of the inguinal lymph node block in two weeks of treatment. Severe neutropenia was diagnosed and complicated with sepsis. The evolution is unfavorable with the installation of multiple organ dysfunction. The presence of a complex karyotype (trisomy 8, trisomy 13) in a patient with myeloid metaplasia with triple-negative PMF was associated with blast transformation and severe thrombocytosis. The patient was diagnosed concomitantly with DLBCL, making the therapeutic decision difficult. Venetoclax has been shown to be useful in the treatment of DLBCL but has been associated with severe neutropenia, which has led to infectious complications.

## Introduction

Large B cell non-Hodgkin's malignant lymphoma (DLBCL) represents 30-40% of the total number of patients diagnosed with non-Hodgkin's lymphoma (NHL). This type of NHL presents two molecular subtypes: germinal center B-cell-like (GCB) and activated B-cell-like (ABC) subtypes; however, 10-15% of cases are unclassifiable [1]. The diagnosis of NHL in rare cases may be associated with chronic myeloproliferative neoplasms (MPNs). Sometimes the diagnosis of MPNs has been established in patients with NHL history during the remission period [2]. Patients with MPNs may have a second neoplasm, with a risk of 1.5 to 3 times for solid tumors (skin, lung, thyroid, kidney) and 2.5 to 3.5 times for lymphoid line neoplasms: monoclonal gammopathy or chronic lymphoproliferative syndrome: B chronic lymphocytic leukemia (B-CLL), NHL [3,4]. The higher incidence of the development of lymphoproliferation in patients with MPNs is higher in men and those with the JAK2V617F mutation and the median duration for the identification of the second neoplasm is 68 months after the diagnosis of MPNs. The risk of lymphoproliferation is three times higher for NHL and up to 12 times for B chronic lymphocytic leukemia (B-CLL). The explanation for this association is the genetic instability of hematopoietic progenitors in MPNs [3]. Some studies have shown that treatment with JAK inhibitors can be involved in increasing the risk of developing NHL, a hypothesis unconfirmed by other studies. However, not all studies have confirmed this hypothesis [5]. The concomitant presence of acute myeloid leukemia (AML) and DLBCL is rarely reported in the literature. Acute myeloid leukemia discovered in DLBCL patients is often secondary to chemotherapy that includes alkylating agents (fludarabine, cyclophosphamide), especially if it also combines rituximab but is also due to immune dysfunction or infections [2,6]. The association at the time of diagnosis of AML and NHL was very rarely reported [7].

We will present the clinical case of a patient diagnosed concomitantly with DLBCL and AML secondary to primary myelofibrosis (PMF) who presented a complex karyotype of trisomy 8 and 13 and triple-negative PMF status.

## Case presentation

**Figure 1:**
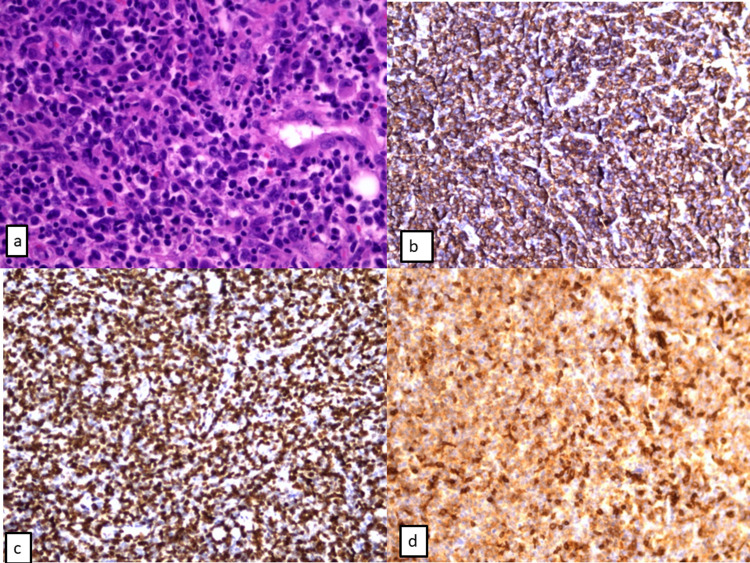
Lymph node biopsy a) diffuse malignant lymphoid proliferation with large-sized cells (HE stain, ob 20x). b) Proliferation is indicated by large B-cells diffusely positive for CD20 (immunohistochemistry [IHC] stain for CD20 Ab, ob 20x); c) the tumor presents a very high (85%) Ki67 proliferation index (IHC stain for Ki67 Ab, ob 20x); d) malignant proliferation of large B-cells presents an aberrant expression of CD5 (IHC stain for CD5 Ab, ob 20x).

The patient is an 80-year-old Caucasian man with known ischemic heart disease, mitral and tricuspid insufficiency, hypertension, heart failure class III NYHA, morbid obesity, presents for physical asthenia, sweating. The patient has not had any AML specific symptoms and has no family history of hematological malignancies or other cancer. The clinical examination shows only right inguinal adenopathy with a diameter of 5-6 cm, partial mobility, and pain (appearing one month before the examination). Laboratory investigation at the onset: white blood cells (WBCs) 3.14 x × 109/L (N 4-11 x × 109/L), hemoglobin (Hb) 10.6g/dl (N 12.3-17 g/dl), platelets (Plt) 1380 x 109/L (N 150-450 x 109/L); peripheral blood smear revealed 2% erythroblasts, 25% blasts, 43% lymphocytes, 14% monocytes, 16% neutrophils, macro platelets and giant platelets, normochromic red cells, frequent polychromatic cells, anisocytosis, ovalocytes and rare teardrop cells. Bone marrow aspiration revealed normal cellularity with 35-40% myeloblasts and very large groups of platelets. Abnormal biochemistry results were uric acid 8.6 mg/dl (N 3.4-7 mg/dl), lactic acid dehydrogenase (LDH) 502 IU/L (N 135-225 IU/L), and C-reactive protein (CRP) 39.72 mg/L (N 0-5 mg/L); the rest of the hematologic, coagulation and biochemistry parameters were normal. It should be noted that the platelet count in January 2021 was 461 x 109/L, and the rest of the hematological parameters were normal. Lymph node biopsy was performed, and the histopathological result identified diffuse malignant lymphoid tumor proliferation with medium/large polymorphic cells, rounded, incised, lobed nuclei, hardly visible nucleoli, and weak basophilic cytoplasm. Immunohistochemistry showed that lymphoid tumor proliferation was B large cell positive diffuse for CD20+ and BCL2, with phenotype CD10 negative, BCL6 positive, and MUM1 positive. The proliferation index Ki67 was 85%, with aberrant expression of CD5 and negative for Cyclin D1, CD3 CD30, and CD23, establishing the diagnosis of DLBCL not otherwise specified (NOS) with a nongerminal center phenotype/non-GCB (Figure 1).

**Figure 2:**
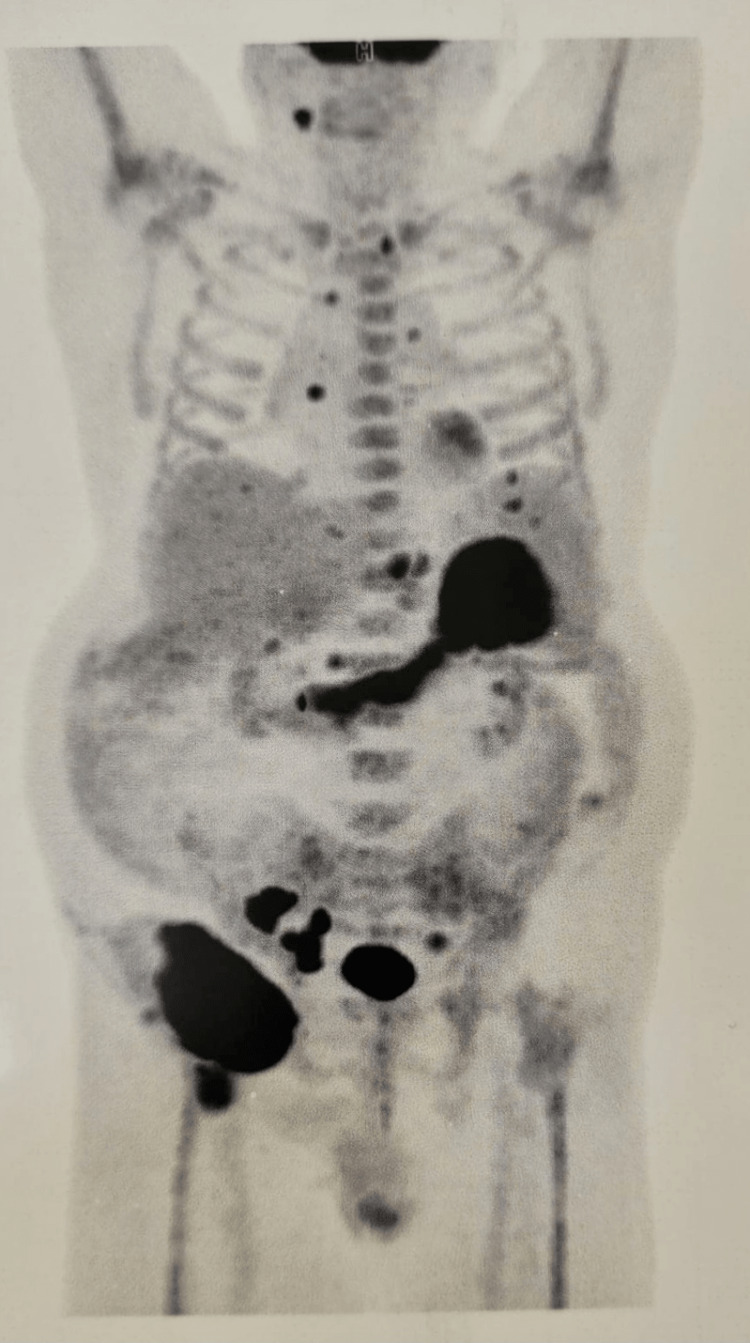
PET-CT result Intense uptake at the level of an inter gastrosplenic tumor mass (maximum standardized uptake value [SUVmax] = 16.13) goes further along the large curvature of the stomach and multiple uptaken lymph nodes situated above and under the diaphragm, isolated or organized as confluated masses, with more expressive masses located at the right inguinofemoral region (SUVmax = 26,08). There was also diffuse and inhomogeneous uptake at the level of the medulla throughout all scanned bones, suggesting myeloproliferative neoplasms (MPNs).

The PET-CT evaluation revealed a hyperactive voluminous area (maximum standardized uptake value [SUVmax] 16.13) corresponding to a tumor mass of 9.68/8.55 cm located inter gastrosplenic and hypercapture along the great gastric curvature up to the antrum, without clear differentiation between these hyperfixations. Multiple hyperfixed areas located right laterocervical (SUV max 9.39 and dimensions 2/1.85 cm), mediastinal (SUV max 7.33), intra-abdominal (celiac area SUV max 7.34, dimensions 2.62/2.35 cm, hilum splenic with confluent appearance and SUV max 5.23, paraaortic and right paravertebral), right internal and external iliac (SUV max 17.11 and dimensions 3.3/2.38cm), left obturator (SUV max 5.82), right inguinofemoral with confluent aspect (SUV max 26.08, diameter 9.97/10.29cm) in correspondence with lymph node formations. Diffuse and inhomogeneous hypertracking of the radiotracer at the bone marrow level of the scanned bone segments suggestive of the presence of MPNs (Figure 2).

**Figure 3:**
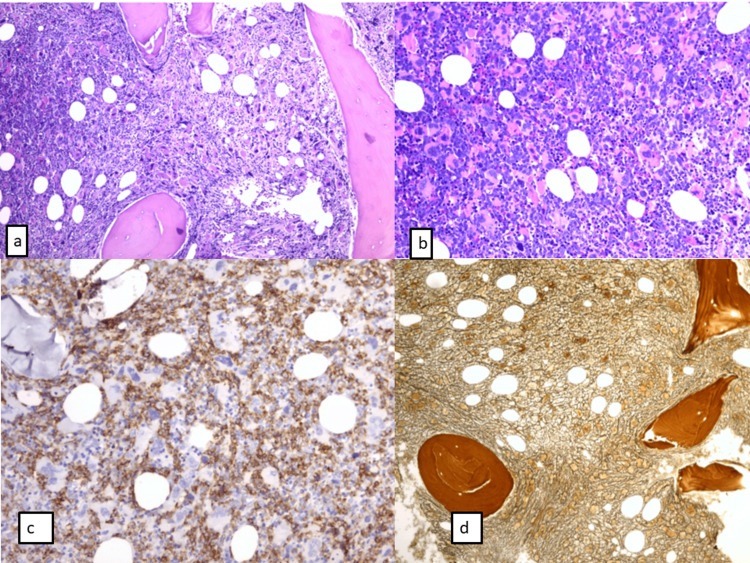
Bone marrow trephyne biopsy a) Hypercellular marrow with granulocyte and megakaryocyte proliferation; areas with dense clusters of atypical megakaryocytes (HE stain, ob 20x); b) Inegal distribution of blasts - areas with clusters of blastic cells (HE stain, ob 20x); c) Blasts are positive for CD34; the percent of CD34 positive blasts is up to 20% of the bine marrow nucleated cells (immunohistochemistry [IHC] stain for CD34 Ab, ob 20x); d) Grade 2 fibrosis (reticulin stain, ob 10x).

Upper digestive endoscopy does not show any pathology. Osteomedullary biopsy (trephine biopsy) performed for staging and diagnosis of DLBCL but also for differential diagnosis of thrombocytosis) indicates the presence of medullary hypercellularity (85%) by diffuse blast infiltration (approximately 50%) with medium cell, vesicular, rounded nucleus, hardly visible nucleoli, normoblasts maturation with rare macro/megaloblastic elements, very common megakaryocytes, very polymorphic, some with nucleocytoplasmic atypia grouped compactly perivascular and per trabecular. Silver impregnation Gomori MF2 focal (>30%). This result is suggestive of acute transformation (AML) of primary myelofibrosis (PMF) (Figure 3).

**Figure 4:**
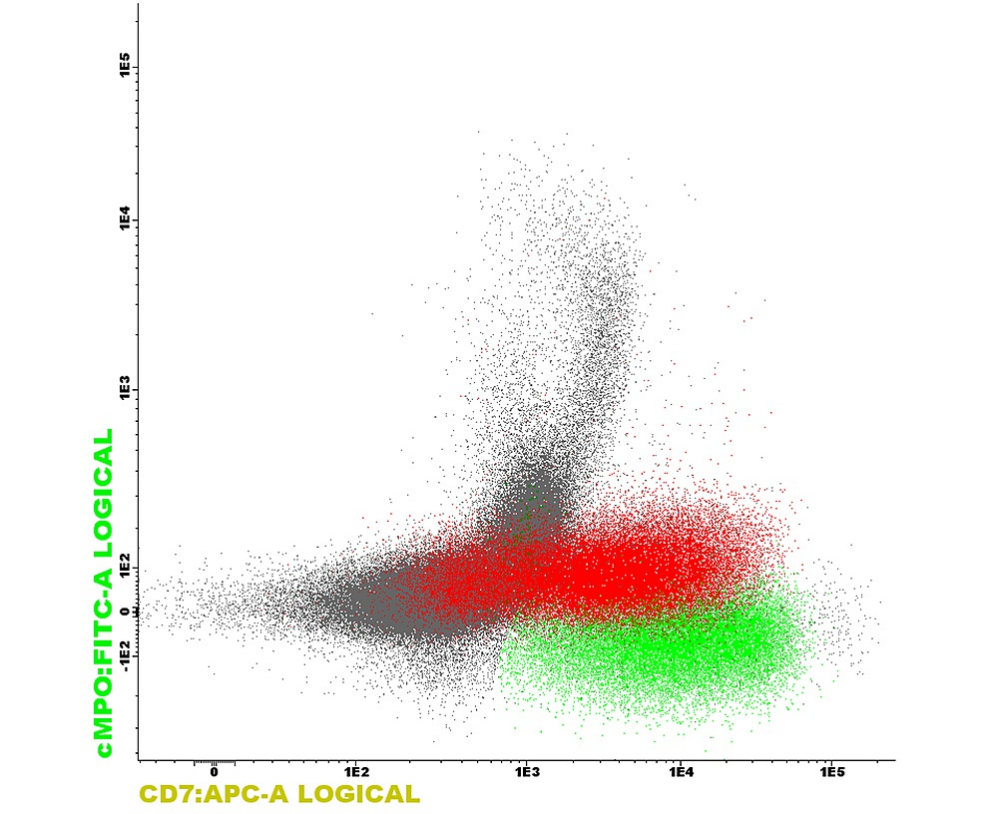
CD7 expression CD7-green - lymphocytes, purple - neutrophils, red - blasts, burgundy - erythroid precursors. The blast population (red) is positive for CD7, internal positive lymphocyte standard (green) and internal negative erythroid series (cherry)

Molecular testing of BCR/ABL, JAK2 V617F, CALR and Mpl was negative. ASXL1 and TET2 mutation was not performed. Immunophenotype evaluation of bone marrow by flow cytometry indicated 30% myeloid blasts CD34 +, CD117 +, HLA-DR +, CD3-, CD7 +, CD19-, CD33 +, CD36-, CD13 +, CD300e-, CD35-, CD14 +/-, CD16-, CD64-, CD11b- , CD10-, CD15-, CD56-, CD9 +/-, CD123 +/-, CD105-, and CD71 +/- suggestive for myeloblast without maturation with aberrant coexpression of CD7, CD9, CD13 (AML M1 FAB) (Figure 4).

Cytogenetic examination of bone marrow aspirate revealed the presence of trisomy 8 and 13. FISH examination did not reveal IGH-BCL2 rearrangement (t (14; 18) (q32, q21) or BCL 6 (3q27). Additionally, MYC rearrangements (8q24) and FGFR1 (8p11) were not revealed. Deletions of the DLEU (13q14), TP53 (17p13), or KMT2A rearrangement (11q23) genes were not detected. The diagnosis was DLBCL stage III in a patient with blastic phase PMF (AML M1 FAB post-PMF) associated with trisomy 8 and 13.

The patient initially received two well-tolerated R mini CHOP series due to a severe clinical picture of DLBCL. This type of treatment was selected to treat DLBCL for one unfit patient for intensive chemotherapy due to his age and comorbidities. R mini CHOP administration was followed by severe aplasia that lasted approximately two weeks followed by severe thrombocytosis that reached 4000 x109/L. For this reason, it was decided to administer anagrelide (Thromboreductin) under which the platelet count returned to normal. Administration of anagrelid was kept in order to maintain a normal platelet count. The result after administration of the two R mini CHOP applications was a partial response with a slight reduction of the right inguinal lymph node block. After one month, the patient presented a deterioration of cardiac function with the effect of myocardial contractility, the appearance of pulmonary hypertension, and valvular-mitral and tricuspid insufficiencies that contributed to the existing complications (ventilatory dysfunction and pleural and pericardial effusions). The decision was made to stop the administration of R mini CHOP (due to severe neutropenia as an adverse event and loss of response by increasing the size of the lymphadenopathy) and to start taking venetoclax. It was selected for the simultaneous treatment of DLBCL and the underlying AML. The evaluation made two weeks after the onset of venetoclax indicated a significant reduction in the size of the inguinal lymph node block as well as the presence of severe leukopenia associated with moderate anemia. During pancytopenia, the patient presents with sepsis with pulmonary origin. CT evaluation shows right lobe pneumonia associated with small bilateral pleural effusion and minimal pericardial effusion. All microbiologic cultures (blood, sputum) were negative, but the patient was positive for severe acute respiratory syndrome coronavirus 2 (SARS-CoV-2), and the rest of the serology tests, cytomegalovirus (CMV) and Epstein-Barr virus (EBV) were negative. At admission, the WBC count was 0.56 x 109/L, Hb 6.5 g/dl, Plt 781 x 109/L, IL-6 84 pg/ml, CRP 136.57 mg/L, procalcitonin 1 ng/mL (N< sau= 0.05 ng/ml), and D dimers 1.43 µg/ml fibrinogen-equivalent units (FEU; N 0-0.5 µg/ml FEU). The outcome is unfavorable with the onset of multiple organ dysfunction, aggravation of renal failure, the onset of severe ventilatory dysfunction, and toxic septic shock. 

## Discussion

The patient was diagnosed with AML M1 FAB (with abnormal expression of CD7) secondary to PMF. The CD7 expression is associated with a poor prognosis of AML [8]. Two genetic aberrations are associated - trisomy 8 and 13. Trisomy 8 is one of the most common cytogenetic mutations found in AML (10-15%) but also in other neoplasms - myelodysplastic syndromes (MDS) (15-20%), MPNs, acute lymphoblastic leukemia (ALL) (5%), solid tumors (colon, breast), rarely in B-CLL or NHL [9-10]. The prognosis of AML 8+ patients is poor; patients with NHL have not been reported [9]. Trisomy 13 has been described in AML M0 and M1 FAB, especially in men over 70 years of age (0.7%), MDS, PMF, and atypical chronic granulocytic leukemia (atypical CML). The prognosis of AML 13+ patients is unfavorable, with a median survival of 0.5-14.7 months [11].

At the same time, the patient was diagnosed with DLBCL NOS with a nongerminal center B cell (GCB) phenotype and aberrant expression of CD5. CD5 expression in DLBCL is more frequently associated with advanced disease or the presence of extranodal determinations. The clinical evolution of such a patient is unfavorable [12]. The concomitant diagnosis of AML and NHL is rare. Cases of AML have been reported in patients with untreated B-CLL, and this association gives a reserved prognosis [13]. In the case of the presented patient, there was no treatment prior to the diagnosis of NHL and transformed MPNs. Although the diagnosis of AML secondary to PMF was made concomitantly with that of NHL-DLBCL, it should be noted that this patient had elevated platelet counts at least six months prior to diagnosis, and the patient was not investigated as a clinical picture or peripheral blood smear. This patient has two trisomy 8 and 13 genetic mutations that give a reserved prognosis. Trisomy 8 has been found, in a few cases, in patients with T cell NHL or B cell NHL (B-CLL, DLBCL, follicular or mantle cell NHL) [14,15]. It has also been identified in patients diagnosed with MDS or MPNs. The unique presence of trisomy 8+ in MDS patients indicates the presence of an intermediate prognosis. However, the association with other anomalies determines the inclusion of the patient in the category of unfavorable prognosis [15]. In our patient, trisomy 8 was associated with trisomy 13, a less common cytogenetic abnormality. Baer reported the presence of trisomy 13 in patients, especially in elderly men, with AML with undifferentiated or biphenotypic type, and the prognosis of these patients was poor [16]. Trisomy 13 was also identified in patients with PMF, and the evolution of these patients was rapid with blastic transformation [17]. The presence of the complex karyotype represented by trisomy 8, 13 and deletions del (20q), del (13q), or chromosome 1 abnormalities are associated with the blastic transformation of PMF and decreased patient survival. The patient presented associated trisomy 8 and 13, explaining the rapid unfavorable evolution. In addition, it is associated with the triple-negative status of PMF, and this status is associated with an increased risk of blast transformation and unfavorable prognosis [18]. The primary triple myelofibrosis should also be differentiated from secondary myelofibrosis secondary to DLBCL or AML. PET CT examination as well as bone marrow HP and IHC examination are important in the differential diagnosis of secondary myelofibrosis due to AML evolution [19]. Both ex HP IHC and ex PET CT performed in our patient confirm the diagnosis of the blastic phase of PMF. In addition, the patient has been diagnosed with aggressive NHL, a recent clinical manifestation. A variety of lymphoproliferative neoplasms may be associated with secondary myelofibrosis (B-CLL, HL or NHL, HCL, multiple myeloma) and secondary fibrosis [20]. The pathogenetic mechanism involved in the onset of secondary myelofibrosis could be the release of IL-1 by neoplastic cells that stimulate fibroblast growth by inducing secondary fibrosis. Another explanation could be the separate proliferation of two clones that are due to molecular and chromosomal abnormalities of the multipotent hematopoietic cell, thus causing bilinear clonal proliferation [20].

## Conclusions

The presence of trisomy 8, trisomy 13 in a patient with myeloid metaplasia with triple-negative PMF was associated with blast transformation and severe thrombocytosis. The patient was diagnosed concomitantly with DLBCL, making the therapeutic decision difficult. Venetoclax has been shown to be useful in the treatment of DLBCL but has been associated with severe neutropenia, which has led to infectious complications.
